# Outcomes of ST Segment Elevation Myocardial Infarction without Standard Modifiable Cardiovascular Risk Factors – Newer Insights from a Prospective Registry in India

**DOI:** 10.5334/gh.1189

**Published:** 2023-03-16

**Authors:** Gnanaraj Justin Paul, Sabarish Sankaran, Karthikaa Saminathan, Mohamed Iliyas, Suryakanth Sethupathy, Sivasubramaniam Saravanan, Salai Sudhan Prabhu, Sijoy Kurian, Sandeep Srinivas, Polavarappu Anurag, Kumaran Srinivasan, Elavarasi Manimegalai, Swaminathan Nagarajan, Rajasekar Ramesh, P. M. Nageswaran, Venkatesan Sangareddi, Ravishankar Govindarajulu

**Affiliations:** 1 Institute of Cardiology, Madras Medical College, Chennai, Tamil Nadu, India; 2 Rajiv Gandhi Government General Hospital, Park Town, Chennai, India; 3 The Tamil Nadu Dr MGR Medical University, Guindy, Chennai, India

**Keywords:** Myocardial infarction, atherosclerosis, risk factors, outcome, mortality

## Abstract

**Objectives:**

Patients with ST elevation myocardial infarction (STEMI) without standard modifiable cardiovascular risk factors (SMuRFs; dyslipidaemia, hypertension, diabetes mellitus and smoking) are reported to have a worse clinical outcome compared to those with SMuRFs. However, robust prospective data and low-and middle-income country perspective are lacking. We aimed to study the patients with first STEMI and assess the influence of SMuRFs on clinical outcomes by comparing the patients with and without SMuRFs.

**Methods:**

We included all consecutive STEMI patients without prior coronary artery disease enrolled in the Madras Medical College STEMI Registry from September 2018 to October 2019. We collected baseline clinical characteristics, revascularisation strategies and clinical outcome. We analysed suboptimal self-reported sleep duration as a 5^th^ extended SMuRF (eSMuRF). Primary outcome was in-hospital mortality. Secondary outcomes included in-hospital complications and one-year all-cause mortality.

**Results:**

Among 2,379 patients, 605 patients (25.4%) were SMuRF-less. More women were SMuRF-less than men (27.1% vs 22.1%; P = 0.012). SMuRF-less patients were older (57.44 ± 13.95 vs 55.68 ± 11.74; P < 0.001), more often former tobacco users (10.4% vs 5.0%; P < 0.001), with more anterior wall MI (62.6% vs 52.1%; P = 0.032). The primary outcome [in-hospital mortality (10.7% vs 11.3%; P = 0.72)] and secondary outcomes [in-hospital complications (29.1% vs 31.7%; P = 0.23) and one-year all-cause mortality (22.3% vs 22.7%; P = 0.85)] were similar in both groups. Addition of suboptimal self-reported sleep duration as a 5^th^ eSMuRF yielded similar results.

**Conclusions:**

25% of first STEMI patients were SMuRF-less. Clinical outcomes of patients without SMuRFs were similar to those with SMuRFs. Suboptimal sleep duration did not account for the risk associated with the SMuRF-less status.

## Introduction

Coronary artery disease is a major cause of death worldwide. Cardiovascular disease is the most common cause of mortality in India, accounting for a third of the certified deaths [1]. Precursors of coronary artery disease (CAD) have been extensively studied and causal risk factors have been identified. The best established modifiable risk factors like hypertension, diabetes, dyslipidaemia and smoking have been the focus of many risk scoring systems for coronary artery disease [2,3,4]. Several risk models that integrate information on conventional cardiovascular risk factors exist [5,6]. The INTERHEART study suggested that nine potentially modifiable risk factors, including diabetes, hypertension, dyslipidaemia and smoking, could account for >90% of population-attributable risk of coronary artery disease [7]. Recognition and management of these risk factors have together led to significant improvements in prevention and therapy [8].

However, it is well known that myocardial infarction also occurs among persons without these traditional risk factors [3]. Contemporary data has shown that as much as 15% to 25% of patients with ST segment elevation myocardial infarction (STEMI) do not have the standard modifiable risk factors (SMuRFs) [9,10,11]. It has been also observed that the proportion of SMuRF-less patients with STEMI is on an increasing trend over the last few decades [12]. Further, many studies have brought out the surprising observation of higher mortality in SMuRF-less patients compared with those with SMuRFs [9,11,12,13]. However, this information comes predominantly from retrospective analyses of studies conducted in high-income nations. Hence, we planned this prospective study to find the proportion of patients with STEMI who are SMuRF-less, compare their in-hospital and intermediate term mortality with those with SMuRFs, and offer a low-and middle-income country perspective.

## Methods

### Data source and study population

Madras Medical College STEMI (M-STEMI) Registry is a prospective registry enrolling acute STEMI patients above 18 years of age seeking care in a public hospital in a metropolitan city in India. All consecutive patients with first diagnosis of STEMI enrolled from September 2018 to October 2019 were included in this analysis after getting their informed consent. Diagnosis of STEMI was based on classic chest pain and diagnostic ST elevation as per standard guidelines [14]. Patients with ST elevation not related to acute coronary syndrome, like Takotsubo cardiomyopathy and acute pericarditis, were excluded. Patients with prior CAD of any form were excluded. The study protocol was designed in accordance with the Code of Ethics of the World Medical Association (Declaration of Helsinki) for experiments involving humans. Written informed consent was obtained from all study participants by the authors.

### Data Collection Instrument

The data collection instrument was developed by the authors in hard copy format convertible into corresponding Microsoft excel tables. The data collection instrument and strategy were put to use in 1,500 consecutive patients admitted with STEMI from October 2017 to August 2018. The data collection instrument and strategy underwent multiple revisions and improvements during this period of trial enrolment of patients to ensure no missing variables. Final data collection was done using a hardcopy of the data collection instrument (Supplementary file-1), which was subsequently updated into an online Microsoft excel spreadsheet by one of the authors.

Baseline demographic factors, cardiovascular risk factors, comorbid conditions and present symptoms and their chronology were collected prospectively for all patients. All patients had detailed clinical evaluation, 12-lead electrocardiogram, echocardiogram and risk stratification on admission. Standard echocardiographic techniques were used to measure left ventricular ejection fraction [15]. Tricuspid annular plane systolic excursion (TAPSE) < 17 mm was used to diagnose right ventricular dysfunction [16]. Management, including revascularisation, was at the discretion of the treating cardiologist. Details of revascularisation modalities, like fibrinolysis, primary percutaneous coronary intervention (PCI), pharmaco-invasive approach or delayed PCI (after 24 hr but before discharge), and details of in-house complications were noted.

### Definitions

Patients who have none of the four standard modifiable cardiovascular risk factors (SMuRFs): hypertension, hyperlipidaemia, diabetes mellitus and current tobacco use, were considered as the SMuRF-less group. Patients with one or more of these four risk factors were considered as the SMuRF-plus group. Current tobacco use was defined as regular use of smoking or non-smoking tobacco for at least the previous one year. Patients who had stopped tobacco use at least 12 months before were labelled as former tobacco users. Considering the high prevalence of smokeless tobacco use in India, we sought for smokeless tobacco use and included it under tobacco use [17]. Hypertension was defined as having an earlier diagnosis of hypertension or prior/current antihypertensive drug therapy. Diabetes was defined as having an earlier diagnosis of diabetes or prior/current use of hypoglycaemic therapy. Dyslipidaemia was defined as having earlier diagnosis of dyslipidaemia or prior/ongoing lipid lowering pharmacologic therapy.

In addition to the four main SMuRFs, suboptimal sleep duration, defined as self-reported sleep duration ≤ 6 hours and > 9 hours, was evaluated as a potential fifth modifiable risk factor. The group with any of these five modifiable risk factors was termed as the extended SMuRF (eSMurRF) group, and the group with none of these as the extended SMuRF-less (eSMurRF-less) group.

### Discharge and follow-up

All patients were discharged with aspirin 150 mg, clopidogrel 75 mg and atorvastatin 80 mg as per protocol, unless contraindicated. All patients were prescribed betablockers and angiotensin converting enzyme inhibitors as permitted by the discharge hemodynamics and biochemistry. Follow-up details were collected at 1, 3, 6 and 12 months. Patients who did not turn up to the outpatient department in time were reminded by telephonic calls by our dedicated and trained follow-up team. Postal letters in vernacular language were sent to the patients who were not reachable by telephone. Our follow-up team performed house visit for the final defaulters.

### Outcome

In-hospital mortality is the primary outcome. The secondary outcomes include a composite of in-hospital complications and one-year all-cause mortality. We are continuing active follow-up of patients.

### Statistical Analysis

Differences between the SMuRF-less group and SMuRF-plus groups in baseline demographics, clinical parameters, reperfusion therapy offered, in-hospital course and complications and follow-up outcome were analysed. An additional similar analysis was performed to find the differences between the eSMuRF and eSMuRF-less groups. Categorical variables are presented as frequencies and percentages and compared using Pearson’s Chi-squared test or Fisher’s Exact Test. Continuous variables are presented as mean, standard deviation (SD) and median (interquartile range), and are compared using student’s t-test (normal distribution) or Mann-Whitney test (non-normal). Covariates with p < 0.10 on univariable analysis were planned to be included in the final multivariable model.

We had a prespecified plan to include any covariates with p < 0.10 on univariate testing in a final multivariate model. Multivariable analysis was done with logistic regression. The results of regression analyses are expressed as an odds ratio (OR) with respective confidence interval (CI) and p-values. Significance was assumed at a two-sided value of p < 0.05. Analyses were performed using SPSS version 28.0 (SPSS for Mac, Version 28.0. Armonk, NY: IBM Corp Released 2021).

## Results

### Study population and baseline features

Between September 2018 to October 2019, 2,499 adults with acute STEMI were enrolled in the M-STEMI registry. Of those, 120 patients with a history of prior CAD were excluded. Of the remaining 2,379 patients with first STEMI analysed, 605 patients with no documented SMuRFs constituted the SMuRF-less group. The rest formed the SMuRFs group. Ninety-nine percent of the study population belonged to the lower socioeconomic category. The Baseline differences in the distribution of demographic variables between the groups are given in Table 1.

**Table 1:** Baseline characteristics of patients with and without SMuRFs – univariable analysis.


VARIABLE	TOTAL (n = 2379)	NO SMURF (n = 605)	≥1 SMURF (n = 1774)	P VALUE

Age				

(Mean age ± SD)	56.13 ± 12.37	57.44 ± 13.95	55.68 ± 11.74	<0.001

<60 years	1392 (58.5%)	323 (53.4%)	1069 (60.3%)	0.003

≥60 years	987 (41.5%)	282 (46.6%)	705 (39.7%)	

Sex				

Male	1823 (76.6%)	441 (72.9%)	1382 (77.9%)	0.012

Female	556 (23.4%)	164 (27.1%)	392 (22.1%)	

Risk Factors				

Hypertension	810	0	810	

Diabetes	933	0	933	

Dyslipidemia	34	0	34	

Current tobacco user	834	0	834	

Former tobacco user	152 (6.4%)	63 (10.4%)	89 (5.0%)	<0.001

Alcohol	838 (35.2%)	113 (18.7%)	725 (40.9%)	<0.001

F/h/o CAD	45	15 (2.5%)	30 (1.7%)	0.219

Sleep duration per day				

Duration (mean ± SD)	7.65 ± 0.89	7.67 ± 0.86	7.64 ± 0.90	0.439

≤ 6 hours	300 (12.6%)	72 (11.9%)	228 (12.9%)	0.810

>6 to ≤ 7 hours	364 (15.3%)	88 (14.5%)	276 (15.6%)	

>7 to ≤ 8 hours	1570 (66%)	411 (67.9%)	1159 (65.3%)	

>8 to ≤ 9 hours	127 (5.3%)	29 (4.8%)	98 (5.5%)	

>9 hours	18 (0.8%)	5 (0.8%)	13 (0.7%)	

CKD	26 (1.1%)	5 (0.8%)	21 (1.2%)	0.465

CVA	42 (1.8%)	4 (0.7%)	38 (2.1%)	0.017

COPD	21 (0.9%)	6 (1%)	15 (0.8%)	0.740


**Figure 1:**
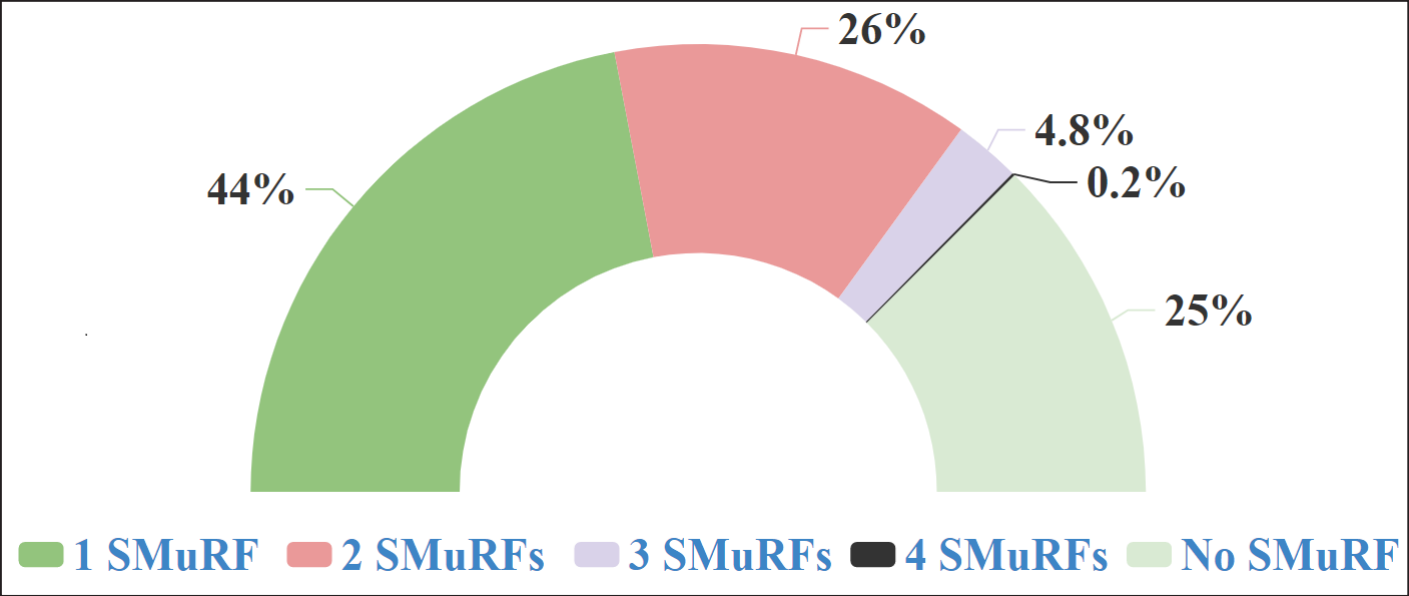
Number of SMuRFS identified in the enrolled patients. *Note*: SMuRF: Standard modifiable cardiovascular risk factor.

The SMuRF-less group had a higher mean age (57.4 vs 55.7%; P < 0.001) with a larger proportion of patients above 60 years of age (46.6% vs 39.7%) compared to the SMuRF-plus group. The study participants were predominantly men (76.6%). However, a larger proportion of women were SMuRF-less compared to men (29.5 % vs 24.2%; P = 0.012). The age and sex differences were not significant in the multivariable analysis. Figure 1 shows the number of SMuRFs present in the study population. The SMuRF-less group had a higher proportion of former tobacco users and lower proportion of ethanol users. Sleep duration was not significantly different between the groups. The proportions of patients with chronic kidney disease (CKD) and chronic obstructive pulmonary disease (COPD) were similar, while the proportion with a cerebro-vascular accident (CVA) was significantly lower in the SMuRF-less group.

### Clinical presentation and reperfusion strategies

The time from symptom onset to presentation in the hospital and presentation Killip class was similar in both the groups. Though a higher proportion of patients in the SMuRF-less group presented with anterior wall MI, the mean left ventricular ejection fraction was similar in both groups (Table 2).

**Table 2:** Clinical presentation and reperfusion strategies.


PARAMETER ANALYZED	TOTAL (n = 2379)	NO SMURF (n = 605)	≥1 SMURF (n = 1774)	P VALUE

Time Window (symptom onset to presentation at hospital)				

Time window (hours)	13.23 ± 17.47	13.08 ± 17.25	13.28 ± 17.25	0.809

<6 hours	1234 (51.9%)	313 (51.7%)	921 (74.6%)	0.794

6–12 hours	534 (22.4%)	143 (23.6%)	391 (22%)	

12–24 hours	315 (13.2%)	79 (13.1%)	236 (13.3%)	

>24 hours	296 (12.4%)	70 (11.6%)	226 (12.7%)	

Location of infarction				

Anterior	1374 (57.8%)	379 (62.6%)	995 (56.1%)	0.005

Non anterior	1005 (42.2%)	226 (37.4%)	779 (43.9%)	

Killip Class				

Class I	1773 (74.5%)	466 (77.0%)	1307 (73.6%)	0.105

Class II, III & IV	606 (25.5%)	139 (23.0%)	467 (23.6%)	

Left ventricular ejection fraction				

Mean EF^a^ ± SD^b^	46.1 ± 8.78	46.13 ± 8.9	46.10 ± 8.45	0.938

≤ 40%	676 (28.4%)	163 (26.9%)	513 (28.9%)	0.643

41–54%	1260 (53.0%)	326 (53.9%)	934 (52.6%)	

>54%	443 (18.6%)	116 (19.2%)	327 (18.4%)	

Right ventricular function				

TAPSE^c^ mean ± SD	17.81 ± 2.46	18.05 ± 2.3	17.74 ± 2.51	0.03

TAPSE < 17	297 (12.5%)	57 (9.4%)	240 (13.5%)	0.008

Primary reperfusion strategy^g^				

Fibrinolysis	1242 (52.2%)	320 (52.9%)	922 (52%)	0.852

SK^d^	1097 (46.1%)	278 (46%)	819 (46.2%)	

TNK^e^	121 (5.1%)	35 (5.8%)	86 (4.8%)	

Reteplase^f^	24 (1.0%)	7 (1.2%)	17 (1.0%)	

Primary PCI	238 (10%)	63 (10.4%)	175 (9.9%)	

Neither	899 (37.8%)	222 (36.7%)	677 (38.2%)	

**Overall Reperfusion Strategyh**				

Primary/PI PCI	354 (14.9%)	90 (14.9%)	264 (14.9%)	0.961

Fibrinolysis only (no PCI)	1035 (43.5%)	266 (44.0%)	769 (43.3%)	

Neither	990 (41.6%)	249 (41.2%)	741 (41.8%)	


^a^EF – Ejection fraction; ^b^SD: Standard deviation; ^c^TAPSE – Tricuspid annular plane systolic excursion; ^d^SK- Streptokinase; ^e^TNK tPA -Tenecteplase; ^f^PCI-Percutaneous coronary intervention; ^g^Primary reperfusion strategy- The type of reperfusion therapy offered at admission to the hospital; hOverall reperfusion strategy: The reperfusion therapy offered during the entire hospital stay.

The proportion of patients with inferior infarction and right ventricular dysfunction was lower in the SMuRF-less group. Fibrinolysis (51%) was the predominant mode of reperfusion, used with only 10% receiving primary PCI. However, the proportion of patients receiving the various modes of reperfusion (Primary PCI, pharmaco-invasive PCI, delayed PCI and standalone fibrinolysis) was similar in both groups.

### Angiographic analysis

Angiographic details were available for 1,089 of the 2,379 patients. LAD involvement was non-significantly higher in the SMuRF-less group. There was no significant difference in the culprit lesion profile and proportion of patients with multivessel disease between both the groups (Table 3).

**Table 3:** **Angiographic analysis.** Analysis of angiographic findings of the 1089 patients who underwent coronary angiogram.


PARAMETER	TOTAL (n = 1089)	NO SMURF (n = 270)	≥1 SMURF (n = 819)	P VALUE

**Culprit Lesions**				

LMCA	3 (0.2%)	1 (0.4%)	2 (0.2%)	0.605

LAD	554 (50.9%)	145 (53.7%)	409 (49.9%)	

LCX	51 (4.7%)	8 (3.0%)	43 (5.3%)	

RCA	218 (20.0%)	55 (20.4%)	163 (19.9%)	

Unspecified	263 (24.2%)	61 (22.6%)	202 (24.7%)	

Single Vessel Disease	508 (46.6%)	127 (47%)	381 (46.5%)	0.883

Multivessel Disease	581 (53.4%)	143 (53%)	438 (53.5%)	


LMCA-Left main coronary artery; LAD- Left anterior descending artery; LCX- Left circumflex coronary artery; RCA-Right coronary artery.

### In-hospital Outcome

Of the 2,379 patients included in the analysis, 265 patients (11.1%) died in the hospital. There was no difference in the in-hospital course or complications between the groups with and without SMuRFs (Table 4). SMuRF-less status did not alter the risk for in-hospital mortality in the stratified analysis done according to age, sex or location of MI (Table 5).

**Table 4:** **In-hospital and 12 months Outcome – univariable analysis.** [Univariate analysis of the difference in the outcome parameters between the SMuRFless and the SMuRF group, expressed as proportions, P value and odds ratio].


PARAMETERS	TOTAL (n = 2379)	NO SMURF (n = 605)	≥1 SMURF (n = 1774)	P VALUE	UNADJUSTED ODDS RATIO (95% CI)

**In-Hospital Outcome**					

In hospital Mortality	265 (11.1%)	65 (10.7%)	200 (11.3%)	0.720	0.96 (0.77–1.20)

Any complications	739 (31.1%)	176 (29.1%)	563 (31.7%)	0.225	0.91 (0.78–1.06)

Arrhythmic complications	589 (24.8%)	132 (21.8%)	457 (25.8%)	0.052	0.85 (0.72–1.0)

Tachyarrhythmia	387 (17.1%)	84 (13.9%)	303 (17.1%)	0.066	0.83 (0.68–1.02)

Bradyarrhythmia	207 (8.7%)	49 (8.1%)	158 (8.9%)	0.543	0.923 (0.72–1.12)

Mechanical Complications	45 (1.9%)	15 (2.5%)	30 (1.7%)	0.219	1.32 (0.87–2.0)

Ventricular septal rupture	37 (1.6%)	13 (2.1%)	24 (1.4%)	0.17	1.39 (0.89–2.12)

Cardiogenic Shock	229 (9.6%)	55 (9.1%)	174 (9.8%)	0.605	0.94 (0.74–1.12)

Right ventricular dysfunction	297 (12.5%)	57 (9.4%)	240 (13.5%)	0.008	(0.73 (0.57–0.93)

**Follow up Outcome**					

Discharged alive	2114 (88.9%)	540 (89.3%)	1574 (88.7%)	0.720	0.96 (0.77–1.20)

Lost to follow up	344 (16.3%)	84 (15.6%)	260 (16.5%)	0.601	0.93 (0.71–1.22)

Post discharge mortality (n = 2035)	194 (9.5%)	51 (9.8%)	143 (9.4%)	0.818	1.03 (0.80–1.32)

One -year mortality (n = 2035)	459 (22.6%)	116 (22.3%)	343 (22.7%)	0.854	0.98 (0.82–1.12)


**Table 5:** **Sub group analysis of In Hospital Mortality and SMuRF less ness.** [Role of the baseline features in modulating the influence of SMuRFless-ness on in-hospital mortality].


OVERALL	≥ 1 SMURF			NO- SMURF		OR	95% CI	P-VALUE FORINTERACTION

		ALIVE N (%)	DEATH N (%)	ALIVE N (%)	DEATH N (%)			

Whole group		1574 (88.7)	200 (11.3)	540 (89.3)	65 (10.7)	0.947	0.704–1.274	0.720*

Age	<60	996 (93.2)	73 (6.8)	308 (95.4)	15 (4.6)	0.690	0.341–1.399	0.304

	>60	578 (82.0)	127 (18.0)	232 (82.3)	50 (17.7)			

Sex	Male	1248 (90.3)	134 (9.7)	405 (91.8)	36 (8.2)	0.788	0.403–1.542	0.487

	Female	326 (83.2)	66 (16.8)	135 (82.3)	29 (17.7)			

Ex Smoking	Yes	79 (88.8)	10 (11.2)	60 (95.2)	3 (4.8)	2.466	0.583–10.441	0.220

	No	1495 (88.7)	190 (11.3)	480 (88.6)	62 (11.4)			

AWMI	Yes	873 (87.7)	122 (12.3)	337 (88.9)	42 (11.1)	1.185	0.576–2.438	0.645

	No	701 (90.1)	78 (10.0)	203 (89.8)	23 (10.2)			

RVD	Yes	188 (78.3)	52 (21.7)	45 (78.9)	12 (21.1)	1.079	0.425–2.742	0.873

	No	1386 (90.4)	148 (9.6)	495 (90.3)	53 (9.7)			

CAR	Yes	355 (77.7)	102 (22.3)	104 (78.8)	28 (21.2)	1.129	0.584–2.184	0.718

	No	1219 (92.6)	98 (7.4)	436 (92.2)	37 (7.8)			


* This is not a p value of interaction. This is the P value of differences between ≥ 1 SMuRF group and SMuRF less group in the entire study cohort. AWMI Anterior wall Myocardial infarction; RVD Right Ventricular dysfunction; CAR Cardiac arrhythmias.

### 12 months outcome

Follow-up information at 12 months was available for 85.5% of the patients included in the study analysis. Of the total, 344 patients were lost to follow-up. The SMuRF status and eSMuRF status of patients who were lost to follow-up and were available for follow-up were similar. (Supplementary Table-1). Of the 2,114 patients discharged alive, there were 194 additional deaths reported by 12 months. Thus, one-year all-cause mortality was 22.6%. Post discharge mortality (9.8% vs 9.4%; P = NS) and all-cause mortality at 12 months (22.3% vs 22.7%, P = NS) were similar in both the groups (Table 4).

### Extended SMuRF analysis

With self-reported suboptimal sleep duration added as a fifth modifiable risk factor, there were 1,851 patients (87.8%) with at least one of the five eSMuRFs and 528 patients (22.2%) without any of these. The eSMURF analysis showed similar results to the main SMuRF analysis, with no significant differences in the in-hospital or 12-month outcome between the groups with and without eSMuRFs (Supplementary Table-2).

## Discussion

**Figure 2:**
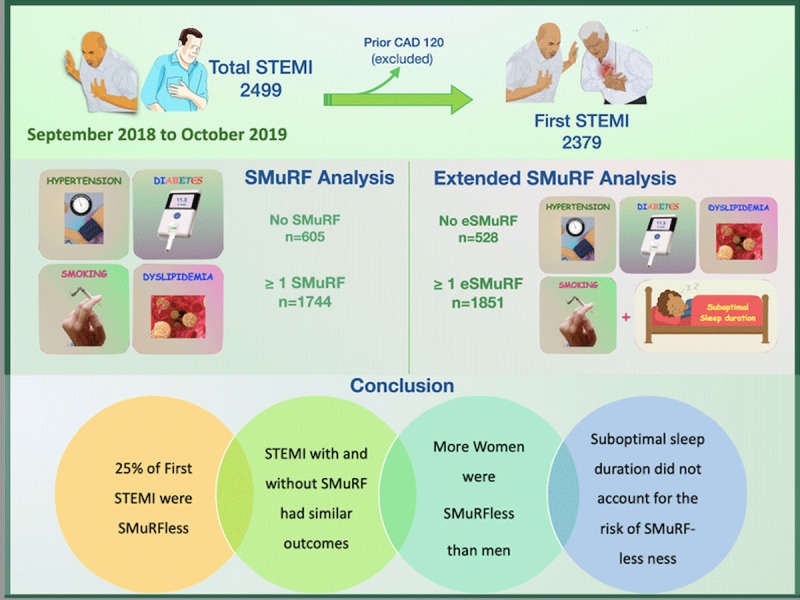
Central illustration-methods and outcome of the study. *Note*: SMuRF—Standard modifiable cardiovascular risk factor; eSMURF—extended standard modifiable cardiovascular risk factor; STEMI—ST segment elevation myocardial infarction.

Our study has four main findings (Figure 2). First, the incidence of STEMI without SMuRFs is high (25.4%) in patients from low-and middle-income countries. Second, the in-hospital mortality, complications and twelve-month mortality in SMuRF-less STEMI patients was similar to those with SMuRFs. Third, more women with STEMI were SMURF-less than men. Fourthly, suboptimal-sleep duration, a recently identified modifiable risk factor, did not account for the risk associated with SMuRFless STEMI.

### Incidence and baseline demographics

The proportion of SmuRF-less STEMI in our study (25.4%) was higher than observed in earlier studies (14.9% & 19%) [11,12]. Since the proportion of SMuRF-lessness in STEMI is likely to vary depending on the vigour at which risk factors are actively looked for in the community, SMuRFs could have been undetected in our study population with poor access to preventive health care, overestimating the SMuRF-less status.

As our study data was collected over 22 months only, we could not comment on the variably reported increasing trend of prevalence of SMuRF-less STEMI over years [9,11,12]. Similar to earlier observations, our patients without SMuRFs were older than those with SMuRFs [11,12]. However, age did not influence the neutral effect of SMuRF-less status on the outcome. Our observation of a higher proportion of STEMI in women being SMuRF-less than in men is different from the earlier observation of SMuRF-less status being more common in men than women [9,11,12]. The reason for this observation is unclear, but potentially hypothesis generating. The possibilities include, but are not limited to, women being less likely to undergo preventive health evaluations [18], and having a higher number of ‘yet-to-be-identified atherosclerotic risk factors’ compared to men, particularly from low-and middle-income countries (LMICs).

### Risk Factors and comorbid conditions

Though the proportion of patients with hypertension, diabetes and current tobacco use in our study was similar to the earlier studies, the proportion of patients with dyslipidaemia was very low. This could be reflective of the unmet needs in diagnosis and management of dyslipidaemia in LMICs [19] compounded by the existing risk factor identification and prevention programmes’ focussing more on hypertension and diabetes than dyslipidaemia [20,21]. Similar to earlier studies, the proportion of former tobacco users was high in the SMuRF-less group. Though we defined former tobacco users as patients who had stopped tobacco use at least 12 months before, it has been shown that the CVD risk remains significantly high in former smokers compared to never smokers for beyond 5 years after quitting [22]. It is possible that former tobacco use could account for some of the risk attributable to the first STEMI in the SMuRF-less patients. The proportion of former tobacco users in our population is low (6.3%) compared to the earlier studies (23% & 27%) [9,11]. This could indicate a smaller contribution from former tobacco use in the atherosclerotic risk of the SMuRF-less group from an LMIC population when compared to developed nations. Though earlier data found comorbid conditions less often in SMuRF-less patients [11], we observed a similar proportion of COPD and CKD in both groups, with only CVA being found in a lower proportion. We did not collect data on obesity, cancer or peripheral vascular disease.

### Clinical presentation and reperfusion strategies

The window period of presentation was similar in both groups, suggesting that being SMuRF-less did not induce delay in seeking medical help. Similar to earlier observations, we observed a higher proportion of patients with anterior wall myocardial infarction and a non-significant higher involvement of LAD as culprit vessel in the SMURF-less group [11,12]. The reasons behind LAD disease being more common in SMuRF-less patients are unclear and open to speculation. As a corollary we observed that inferior wall infarction, and consequently RV dysfunction, was more common in the SMuRF group.

### Revascularisation

Only 10% of our patients underwent primary PCI in our study. Though this number is low, it is not different from earlier reported data from low- and middle-income countries [23,24,25]. However, the proportion of patients receiving various modes of reperfusion therapy (primary PCI, fibrinolysis, pharmaco-invasive therapy, delayed PCI and no revascularisation) was similar between both the groups and hence is unlikely to influence the conclusions of the study.

### Outcome

Our study showed that in-hospital mortality was equal in both the groups with and without SMuRFs. Mortality in acute coronary syndromes has been reported to be worse in SMuRF-less patients compared to those with SMuRFs [11,12,13,26]. Vernon et al. observed a high in-hospital mortality in the SMuRF-less group, however, with similar rates of major adverse cardiac events, cardiogenic shock and in-hospital reinfarctions, and suggested that the reason for the observed increased mortality needs to be investigated further [12]. The study by Figtree et al. also found higher in-hospital mortality, with similar rates of reinfarctions and heart failure in the SMuRF-less group. In the absence of data on cardiac arrhythmia, they had suggested cardiac arrhythmia as a possible contributor of this increased mortality in SmuRF-less group.

Our study, with a similar proportion of cardiac arrhythmia in both the groups, did not support this postulation. Though we found that SMuRF-less status was more common in women, mortality was similar in women with and without SMuRFs, unlike earlier observations [11]. The information obtained from this prospective study does not contradict the message of the earlier studies, but rather confirms the fact that absence of traditional risk factor does not imply good outcome. The adverse outcome in patients without SMuRFs may be because they harbour unidentified/quantified risk factors and they lack a therapeutic target, where ironically the SMuRFs group has an advantage.

### eSMuRF

Association between reduced self-reported sleep duration and coronary artery disease and adverse outcome [27,28,29] has been reported recently. The distribution of sleeping hours was equal in both the groups in our study. The additional eSMuRF analysis, with suboptimal self-reported sleep duration added as the fifth modifiable risk factor, yielded results similar to the main outcome. Earlier observations have suggested that STEMI in the SMuRF-less group could not be explained by obesity and family history of premature atherosclerotic coronary artery disease [12]. This study adds information that this finding could not be explained by suboptimal sleep duration either. The role of non-conventional and lesser studied risk factors, like lipoprotein(a), high-sensitivity C-reactive protein, psychosocial risk factors, access to preventive health care and education, air particulate matter, etc. in contributing to the risk in SMuRF-less STEMI needs further evaluation.

### Strengths and limitations

Our study has the strength of being a large prospective study, evaluating the role of SMuRF-less status in STEMI. This study also evaluated the role of suboptimal sleep duration as an additional fifth modifiable cardiovascular risk factor in STEMI. Our results are not generalisable to populations with improved and widespread preventive health care availability. Our study has several limitations. Data on potential confounders, such as baseline, in-hospital and discharge pharmacotherapy, history of malignancy and peripheral occlusive vascular disease, were not routinely collected and hence could not be analysed as covariates. Data on risk factors like family history of premature coronary artery disease, body weight, body mass index, waist circumference, HBA1C, lipoprotein (a), high-sensitivity C-reactive protein, socio-cultural factors, or psychosocial risk factors were not available. Information on possible differences in access to or use of preventive healthcare was not available. Though the relationship between the risk factors and MI is loglinear, with no identified threshold above which the likelihood of MI increases, we chose to stick with the conventional definitions of the SMuRFs with thresholds and specific cut offs. This helped us to categorise them to two different groups for comparison purpose. However, we acknowledge the global cardiovascular risk assessment should ideally consider the linear relationship of different risk factors with morbidity and mortality outcomes. Coronary angiogram was not done for all patients, bringing in a possibility of our population having patients with spontaneous coronary artery dissection (SCAD) and myocardial infarction with non-obstructive coronary arteries (MINOCA) as a potential limitation. Twelve-month follow-up data was available for only 85.5% of the study participants. Though we could capture the follow-up event data, the date of event was not available for all patients due to the COVID-19 pandemic. Hence, the follow-up outcome could not be presented in a Cox regression (survival) model.

## Conclusion

We observed that one-fourth of patients with STEMI were SMuRF-less. More women were SMuRF-less than men. The clinical outcomes of patients with STEMI without SMuRFs was similar to those with SMuRFs, highlighting that being SMuRF-less does not necessarily confer a lower risk in STEMI. This underscores the need for evidence based on timely revascularisation therapy and pharmaco-therapy for both patients with and without SMuRFs, and the need for studies to evaluate the role of non-conventional and yet-to-be-identified risk factors in STEMI.

## Data Accessibility Statement

The deidentified data underlying this article can be shared on reasonable request to the corresponding author. However, data shall be shared after approval from the Institutional Ethics Committee of Madras Medical College.

## Additional Files

The additional files for this article can be found as follows:

10.5334/gh.1189.s1 Supplementary File 1. Madras Medical College Stemi (M-Stemi) Registry – Proforma.

10.5334/gh.1189.s2 Supplementary Table-1. Baseline characteristics of patients lost to follow up.

10.5334/gh.1189.s3 Supplementary Table-2. Extended SMuRF analysis.

## Figures and Tables

**Table:** 

1	STEMI	:	ST segment Elevation Myocardial Infarction
2	SMuRF	:	Standard Modifiable Cardiovascular Risk Factors
3	eSMuRF	:	Extended Standard Modifiable Cardiovascular Risk Factors
4	CAD	:	Coronary Artery Disease
5	PCI	:	Percutaneous Coronary Intervention
6	COPD	:	Chronic obstructive pulmonary disease
7	CVA	:	Cerebro vascular accident
8	CKD	:	Chronic Kidney Disease
9	LAD	:	Left Anterior Descending Artery
10	LMIC	:	Low-and middle-income countries
