# Supplementary material for: Outcomes of ST Segment Elevation Myocardial Infarction without Standard Modifiable Cardiovascular Risk Factors – Newer Insights from a Prospective Registry in India
Source: Glob Heart. 2023 Mar 16;18(1):13. doi: 10.5334/gh.1189 (PMC10022543; doi:10.5334/gh.1189)
Supplement: Supplementary File 1. — Madras Medical College Stemi (M-Stemi) Registry – Proforma. [file gh-18-1-1189-s1.pdf]

MADRAS MEDICAL COLLEGE STEMI (M-STEMI) REGISTRY - PROFORMA

INSTITUTE OF CARDIOLOGY

MADRAS MEDICAL COLLEGE & RAJIV GANDHI GOVERNMENT GENERAL HOSPITAL, CHENNAI

A. REGISTRATION DETAILS

STEMI Number :

IP Number:

Name	
Age	
Sex	
DOA	
Occupation	
Marital status	
Socioeconomic status	

Date of Admission	
**Address:**	
Phone No 1	
Phone No 2	
Phone No 3	

B. PRESENTING SYMPTOMS

(If yes, mention the duration and remarks if any)

**Symptom**	**Yes/No**	**Duration**	**Remarks**
Chest pain			
SOB			
Palpitation			
Sweating			
Dizziness			
Syncope			
Pre infarction angina			

Time of symptom onset	
Time when patient decided to seek medical help	
Time of first medical contact	
Mode of transport to hospital (Tick the right)	Ambulance / public transport / private / self

C. RISK FACTORS (MODIFIABLE AND NON MODIFIABLE)

(If yes, mention remarks as appropriate)

**Risk Factors**	Yes/ No	Remarks (Details of risk factor, duration, treatment etc)
Diabetes		
SHTN		
CKD		
COPD		
PAD		
Prior CAD		
Dyslipidemia		
Family h/0 CAD		

**Risk Factors**	Yes / No	Remarks (Current / ex / never / quantity, duration)
Smoking tobacco		
Chewable tobacco		
Alcohol		
Substance use		
Psychosocial stress		Anxiety, Depression, Unforgiveness, others

D. PAST HISTORY (If Yes please provide details)

COPD	Yes /No	
CKD	Yes /No	
CVA	Yes /No	

PRIOR CAD: Yes / No

(If yes give details below)

Type of prior CAD	Date, type, location, LV EF, treatment offered like lysis, PCI, CABG etc
SIHD	
NSTEMI	
STEMI	

E. PRESENT STEMI INITIAL RISK ASSESSMENT:

TIMI Risk Score			KILLIP Class	

F. REPERFUSION STRATEGY USED *(DATE, TIME, DETAILS)*

Fibrinolysis:	Pharmaco-invasive PCI:
Primary PCI:	Delayed PCI:

G. Present STEMI ECG ANALYSIS

ECG localisation of IRA. (Tick the appropriate)

LAD	Before S1 & D1 / After S1 before D1/ After D1 before S1/ After S1&D1/ distal /selective D1		
RCA	Proximal RCA before RV branches/ distal RCA/ very dominant RCA		
LCX	Proximal LCX Before OM/OM1 Occlusion/very dominant LCX		
Total (Sigma) ST Elevation		Total (Sigma) mm ST Depression	

H. Present STEMI ECHO EVALUATION. Date:

RWMA				
LVEF			E/e’	
LVDD / LVSD			MR / PMD	
TAPSE			TR	
TRPG			Pericardial effusion	
Free Wall Rupture				
Ventricular septal rupture				
Papillary muscle rupture				
LV Thrombus				
LV Aneurysm				
Others				

I. COMPLICATIONS IN HOSPITAL

ARRYTHMIC COMPLICATION Details with date, type, treatment and outcome

Bradycardia	
Tachycardia	
Bundle branch block	
Others	

MECHANICAL COMPLICATIONS If yes, give details

Cardiogenic shock:	PMD / PMR:
VSR:	FWR:

J. CORONARY ANGIOGRAM

Date of Angiogram:

CULPRIT LESION (Circle correct one)	LMCA	LAD	LCX	RCA	RAMUS	UNCLEAR

Angiography report

LMCA	
LCA	
LCX	
RCA	
IMPRESSION	
PLAN	

K. **PROCEDURE DONE DETAILS**

**Procedure**	Remarks (done /not done, details and outcome
PCI	
CABG	
Others	

L. **HEMODYNAMIC SUPPORT (Remarks, duration, course)**

INOTROPIC SUPPORT	
VENTILLATORY SUPPORT	

M. **FINAL OUTCOME:**

CARDIAC ARREST			
DEATH			
DISCHARGE			

N. **FOLLOW UP DETAILS**

(Status- Alive/Death, Functional class, Repeat Hospitalization (cardiac/non cardiac), Details of ECG and ECHO report are done if any)

1 Month	
3 Month	
6 Month	
1 Year	

==
