# Supplementary material for: Outcomes of ST Segment Elevation Myocardial Infarction without Standard Modifiable Cardiovascular Risk Factors – Newer Insights from a Prospective Registry in India
Source: Glob Heart. 2023 Mar 16;18(1):13. doi: 10.5334/gh.1189 (PMC10022543; doi:10.5334/gh.1189)
Supplement: Supplementary Table-1. — Baseline characteristics of patients lost to follow up. [file gh-18-1-1189-s2.pdf]

Supplementary Table-1: Baseline characteristics of patients lost to follow up

Caption: Univariate analysis results of the SMuRF status and extended SMuRF status of patients who were lost to follow up and who could be followed up at one year

**Variable**	**Discharged alive** (n=2114)	**Followed up** (n=1770)	**Lost follow up** (n=344)	**P value**
SMuRF status				
SMURF less	540 (25.5%)	456 (25.8%)	84 (24.4%)	
SMuRF plus	1574 (74.5%)	1314 (74.2%)	260 (75.6%)	0.601
eSMuRF status				
eSMURF less	470 (22.2%)	392 (22.1%)	78 (22.7%)	
eSMuRF plus	1644 (77.8%)	1378 (77.9%)	266 (77.3%)	0.830
Past Tobacco use	139 (6.6%)	110 (6.2%)	29 (8.4%)	0.129
Sleep duration per day				
Sleep hours (mean ± SD)	7.65± 0.9	7.65 ± 0.9	7.68 ± 0.92	0.309
≤ 6 hours	266 (12.6%)	230 (13%)	36 (10.5%)	
> 6 to ≤ 7 hours	317 (15%)	269 (15.2%)	48 14.0%)	
> 7 to ≤ 8 hours	1399 (66.2%)	1162 (65.6%)	2378.9%)	
> 8 to ≤ 9 hours	116 (5.5%)	95 (5.4%)	21 (6.1%)	
> 9 hours	16 (0.8%)	14 (0.8%)	2 (0.6%)	0.614
