# Supplementary material for: Outcomes of ST Segment Elevation Myocardial Infarction without Standard Modifiable Cardiovascular Risk Factors – Newer Insights from a Prospective Registry in India
Source: Glob Heart. 2023 Mar 16;18(1):13. doi: 10.5334/gh.1189 (PMC10022543; doi:10.5334/gh.1189)
Supplement: Supplementary Table-2. — Extended SMuRF analysis. [file gh-18-1-1189-s3.pdf]

Supplementary Table-2: Extended SMuRF analysis

Caption: Univariate analysis of in-hospital and one-year outcome, done with suboptimal sleep duration added as the 5th modifiable risk factor (eSMuRF)

Variable	Total (n=2379)	No eSMuRF (n=528)	≥ 1 SMuRF (n=1851)	P value	Unadjusted Odds Ratio (95% CI)
In hospital Outcomes					
In hospital Mortality	265 (11.1%)	58 (11.0%)	207 (11.2%)	0.898	0.99 (0.96-1.03)
Any complications	739 (31.1%)	153 (29%)	586 (31.7%)	0.240	0.96 (0.90-1.02)
Arrhythmic complications	589 (24.8%)	114 (21.6%)	475 (25.7%)	0.056	0.95 (0.90-0.99)
Mechanical Complications	45 (1.9%)	13 (2.5%)	32 (1.7%)	0.275	1.01 (0.99-1.02)
Cardiogenic Shock	229 (9.6%)	49 (9.3%)	180 (9.7%)	0.760	0.99 (0.97-1.02)
RV dysfunction	297 (12.5%)	50 (9.5%)	247 (13.3%)	0.018	0.96 (0.93-0.99)
One-year outcome	Total (n=2035)	No eSMuRF (n=450)	≥ 1 eSMuRF (n=1584)	P value	Unadjusted Odds Ratio (95% CI)
Post Discharge Mortality	194 (9.5%)	45 (10%)	149 (9.4%)	0.702	1.05(0.81-1.38)
One-year mortality	459 (22.5%)	103 (22.9%)	356 (22.5%)	0.848	1.02 (0.79-1.31)
